# Public Opinion and Expectations: Development of Public Health Education in China After COVID-19 Pandemic

**DOI:** 10.3389/fpubh.2021.702146

**Published:** 2021-08-10

**Authors:** Xin Shen, Crystal Jingru Li, Tianyi Dong, Hui Cao, Jing Feng, Zihui Lei, Zijian Wang, Xiaotong Han, Chuanzhu Lv, Yong Gan

**Affiliations:** ^1^Department of Social Medicine and Health Management, School of Public Health, Tongji Medical College, Huazhong University of Science and Technology, Wuhan, China; ^2^Department of Psychology, School of Education and Human Development, Hong Kong Education University, Hong Kong, China; ^3^Department of Labor and Environmental Health, School of Public Health, Tongji Medical College, Huazhong University of Science and Technology, Wuhan, China; ^4^Department of Labor Economics and Management, Beijing Vocational College of Labour and Social Security, Beijing, China; ^5^School of Arts and Communication, Guangxi University of Science and Technology, Liuzhou, China; ^6^Department of Emergency Medicine, Hunan Provincial Institute of Emergency Medicine, Hunan Provincial Key Laboratory of Emergency and Critical Care Metabolomics, Hunan Provincial People's Hospital/The First Affiliated Hospital, Hunan Normal University, Changsha, Hunan, China; ^7^Emergency Medicine Center, Sichuan Provincial People's Hospital, University of Electronic Science and Technology of China, Chengdu, China; ^8^Research Unit of Island Emergency Medicine, Chinese Academy of Medical Sciences (No. 2019RU013), Hainan Medical University, Haikou, China; ^9^Key Laboratory of Emergency and Trauma of Ministry of Education, Hainan Medical University, Haikou, China

**Keywords:** public opinion, public health education, China, COVID-19, policy

## Abstract

**Background:** Policymakers must promote the development of public health education and human resources. As a feature of the political environment, public opinion is essential for policy-making, but virtually the attitudes of Chinese citizens toward human resources development in public health is unknown.

**Methods:** This study conducted a crosssectional survey from February 4, 2021 to February 26, 2021 in China. We adopted a convenient sampling strategy to recruit participators. Participants filled out the questions, which assess the attitudes of the expanding public health professionals. A logistic regression analysis was given to identify the predictors associated with the attitudes of the subjects.

**Results:** There were 2,361 residents who have finished our questionnaire. Chinese residents who lived in urban (OR = 1.293, 95% CI = 1.051–1.591), “themselves or relatives and friends have participated in relevant epidemic prevention work” (OR = 1.553, 95% CI = 1.160–2.079), “themselves or family members engaged in medical-related work” (OR = 1.468, 95% CI = 1.048–2.056), and those who “were aware of public health before the outbreak of COVID-19” (OR = 1.428, 95% CI = 1.125–1.812) were more likely to support the promotion of public health education and training.

**Conclusions:** The present study found that 74.50% of Chinese citizens supported the promotion of public health education and training in China, in which economic status, personal perception, and comprehension are the crucial factors that influence public opinion. COVID-19 has aroused the attention of Chinese residents to public health education, with only 22.11% of residents being aware of public health before the outbreak of COVID-19. The COVID-19 pandemic has profound implications for human society. Literally, this impact will feed back into future public health policies based on public opinion. This innovative perspective will also help us better understand the potential social impact of COVID-19 on human resources and development for health in the modern world.

## Background

In recent years, China has experienced many sudden public health events characterized by rapid outbreak, wide spread, and serious damage, such as severe acute respiratory syndrome, the H1N1 flu epidemic, and the coronavirus disease 2019 (COVID-19). These epidemics posed an unprecedented threat to the physical and mental health of the population and to the stability and order of the society (1). Chinese government could cope with multiple crises by relying on a team of public health professionals with rich theoretical knowledge and practical experience (2). However, during the COVID-19 outbreak in 2020, China was faced with a noticeable shortage of public health professionals (3, 4).

This shortcoming has two sides. Firstly, the shortfall is in the personnel size. It is stated in the Outline of the National Health Service System Plan that there must be 0.83 public health personnel per 1,000 permanent residents, but currently, the figure just reached 0.61 (5). According to the China Health Statistical Yearbook in 2018, only 3% of the physicians in China worked in public health services, that is about 114,000 doctors, which is far fewer than clinicians (2.7 million). In terms of educational level, more than half (54%) of the professionals engaged in public health only have the bachelor's degree; just 7% of them have the master's degree (6).

In addition, China faces a shortage of public health training. Inadequate vocational funding and supplies for professional training delay the development and improvement of public health capabilities (7). According to the latest data from the Association of Schools and Programs of Public Health (ASPPH), 61,453 public health students were trained by accredited institutions in 2018, of whom 37% were undergraduates, 49% were masters students, and 14% were doctoral students (8). As of 2020, 77 universities offered public health programmers and 46 universities t were authorized to admit Master of Public Health (MPH) students, with an annual enrollment of about 6,500 (9). Despite the fact that China has established a certification program for public health students, only 60,000 students pass the public health medical practitioner test each year (9). In general, there is a shortage of public health training. Moreover, over the years, the capacity to deal with public health emergencies related to epidemics outbreak is still considered a non-essential training (10). Lack of regular public health emergency training for health care workers contributed to inadequate preparedness and response to the initial COVID-19 outbreak. In brief, the cultivation of public health professionals in China still needs to be paid more attention.

Therefore, policymakers must promote public health education and human resources development, including creating more comprehensive courses on emergency management and expanding the number of public health professionals (11, 12). However, whether policymakers can respond rapidly according to the reality of the situations is still a question worth studying. In terms of the dynamics of policymaking, public opinions will play an essential role in the driving policy. Public opinion can promote the formation of public services and the formulation of health policies by providing support for services that the government or public administrations lack political interest (13). This influence can even extend to the legislative policy (14, 15). Political science research demonstrates that public opinion influences behaviors of the elected policymakers (16–18). The main reason is that the policymakers are motivated by pubic approval and act in ways that they believe are in line with the desires of their constituents (19). Thus, if policymakers understand that the public expects evidence to support their decisions, this information could potentially motivate policy makers and the management departments to make more decisions in line with public opinion and show their constituents the evidence (20).

Although public opinion cannot wholly control the training of public health professionals, in the future, it will still play an essential role in the policy-making process in China. Despite recognition that public opinion and evidence-based decision making will motivate the development of health policies (21), virtually the attitudes of Chinese residents toward the development of human resources for public health is still unknown. As the first country to suffer from COVID-19 and a representative developing country, the survey of public opinion on the development of public health professionals in China can provide references for policymakers in China and the entire world. This innovative perspective will also help us better understand the potential social impact of COVID-19 on human resources and development for health in the modern world.

## Methods

### Study Participants and Survey Design

A crosssectional survey was conducted in China from February 4, 2021 to February 26, 2021. A convenience sampling strategy was adopted to recruit participants; the research team used WeChat (the most popular social media platform in China) to advertise and circulate the survey link to their network members. Network members were requested to distribute the survey invitation to all their contacts. Respondents were stratified according to the eastern (Beijing, Tianjin, Hebei, Liaoning, Shanghai, Jiangsu, Zhejiang, Fujian, Shandong, Guangdong, and Hainan), central (Shanxi, Jilin, Heilongjiang, Anhui, Jiangxi, Henan, Hubei, and Hunan) and western (Chongqing, Sichuan, Guizhou, Yunnan, Tibet, Shaanxi, Gansu, Qinghai, Ningxia, Xinjiang, Inner Mongolia, and Guangxi) regions of China. Participants were informed that their participation was voluntary, and consent was implied by completing the questionnaire. The inclusion criteria were as follows: (1) Chinese citizens who were at least 18 years old and (2) able to comprehend and read Chinese. In our study, a 95% confidence level and ±5% precision are assumed for the Equation.


$$\begin{array}{l} n=\frac{N}{1+N{\left(e\right)}^{2}} \end{array}$$

where *n* is the sample size, *N* is the population size, and *e* is the level of precision. Thus, the conservative total sample size for this questionnaire is 1,200.

### Instruments

The survey consisted of questions that assessed:

1. Socio-demographic characteristics, with seven items, including gender, age, highest educational level, place of residence, religion, and employment status.
2. Personal perception of COVID-19, with four items, including “you or your relative or friend has experienced COVID-19,” “you or your relative or friend has participated in relevant work to prevent epidemic,” “you or your family member is engaged in medical-related work,” and “you were aware of public health before the COVID-19 outbreak.”
3. Attitudes of developing public health professionals, with one item, was “supporting the promotion of public health education and training.” The concept and role of public health are noted in each questionnaire to ensure that participants have a unified understanding.

Wenjuanxing (www.wjx.cn), a widely used platform for conducting surveys in China, developed the electronic questionnaire. An online poster with an access code or the website link to the questionnaire was distributed *via two* ways: (1) we leveraged WeChat (largest messaging platform of China with nearly one billion users, similar to WhatsApp in Western countries) to send the hyperlink of the online questionnaire and (2) distributed *via* WeChat groups, with an average of one to two RMB each as compensation. Each individual could only participate once on each WeChat account to avoid repeated submissions.

### Statistical Methods

The data were analyzed using *SPSS*™ for Windows, Version 22.0 (SPSS, Inc., Chicago, IL, USA). We dichotomized the answers to the attitudes of the residents of supporting the development of public health professionals as “Yes” and “No.” The descriptive statistics was presented as the number of observations with percentage (%), and we analyzed the difference in demographic statistics by Chi-square (χ^2^) test. Due to the disparities in socioeconomic status in different regions, the data have a typical hierarchical structure. We performed a mixed-effect logistic regression model with a random cluster effect (geographic regions) to investigate the adjusted OR (95% CI) of influencing factors of the attitudes of the residents of supporting the development of public health professionals. Further, we explored the factors influencing attitudes of the participants in Eastern, Central, and Western China, respectively, through multivariable logistic regression analysis. The significance level was accepted when *P* < 0.05 (two-sided).

## Results

### Descriptive Statistics

A total of 2,453 residents received the questionnaire, of which 21 participants did not respond and 71 questionnaires were not filled. The response rate was 96.24%, and 2,361 complete questionnaires were employed for results analysis. Table 1 reports the social–demographic characteristics of 2,361 respondents. The mean age was 29.72 years (SD = 6.94), and most of the respondents were female (60.10%). Among the respondents, 421 (17.83%), 1,470 (62.26%), and 470 (19.91%) were from eastern, central, and western China, respectively. Most respondents (89.24%) have a bachelor's degree or higher. More than half of the participants were unemployed (57.05%) and lived in urban (58.11%).

**Table 1:** Statistical description of study samples: univariate analysis of the differences of residents' attitudes of developing public health professionals.

**Variables**	***N* (%)**	***χ2***	***P***
Total	2,361 (100)	NA	NA
Supporting the promotion of public health education and training			
Yes	1,759 (74.50)	NA	NA
No	602 (25.50)		
Gender			
Male	942 (39.90)	2.747	0.097
Female	1,419 (61.10)		
Age group, y			
18–44	1,845 (78.14)	2.168	0.338
45–59	369 (15.63)		
>60	111 (4.70)		
Place of residence			
Urban	1,372 (58.11)	8.705	0.003
Rural	989 (41.89)		
Highest educational level			
Primary school or below	68 (2.88)	2.584	0.275
Middle school	186 (7.88)		
College degree or above	2,107 (89.24)		
Region			
Eastern China	421 (17.83)	8.399	0.015
Central China	1,470 (62.26)		
Western China	470 (19.91)		
Employment status			
Employed	1,014 (42.95)	0.829	0.362
Unemployed	1,347 (57.05)		
You or your relative or friend has experienced COVID-19			
Yes	206 (8.73)	0.018	0.892
No	2,155 (91.27)		
You or your relative or friend has participated in relevant work of prevention epidemic			
Yes	338 (14.32)	8.944	0.003
No	2,032 (85.68)		
You or your family member engaged in medical-related work			
Yes	239 (10.12)	4.762	0.029
No	2,122 (89.88)		
You were aware of public health before the COVID-19 outbreak			
Yes	522 (22.11)	8.818	0.003
No	1,839 (77.89)		

Of them, 1,759 (74.50%) supported the promotion of public health education and training.

Univariate analysis results suggested some statistical factors, such as the place of residence, region, whether “you or your relative or friend has participated in relevant work of prevention epidemic,” “you or your family member was engaged in medical-related work,” and “you were aware of public health before the COVID-19 outbreak” that have a significant influence on “supporting the promotion of public health education and training” (*P* < 0.05; Table 1). Considering the significant differences in geographic regions in the sampling, we respectively conducted univariate analyses with participants from Eastern, Central, and Western China (Table 2).

**Table 2:** Univariate analysis of the differences in attitudes of developing public health professionals among the included residents stratified by geographic characteristics.

**Variables**	**Eastern China**			**Central China**			**Western China**		
	***N* (%)**	***χ2***	***P***	***N* (%)**	***χ2***	***P***	***N* (%)**	***χ2***	***P***
Total	421 (100)	NA	NA	1,470 (100)	NA	NA	470 (100)	NA	NA
Supporting the promotion of public health education and training									
Yes	333 (79.10)	NA	NA	1,067 (72.59)	NA	NA	359 (76.38)	NA	NA
No	88 (20.90)			403 (27.41)			111 (23.62)		
Gender									
Male	181 (42.99)	0.197	0.657	558 (37.96)	0.923	0.337	203 (43.19)	1.698	0.193
Female	240 (57.01)			912 (62.04)			267 (56.81)		
Age group, y									
18–44	323 (76.72)	1.023	0.600	1,175 (79.93)	1.595	0.450	347 (73.83)	0.084	0.959
45–59	61 (14.49)			232 (15.78)			76 (16.17)		
>60	37 (8.79)			63 (4.29)			47 (10.00)		
Place of residence									
Urban	288 (68.41)	4.469	**0.035**	754 (51.29)	2.960	0.085	330 (70.21)	0.486	0.486
Rural	133 (31.59)			716 (48.71)			140 (29.79)		
Highest educational level									
Primary school or below	17 (4.04)	2.224	0.329	27 (1.84)	0.492	0.782	24 (5.11)	1.951	0.377
Middle school	24 (5.70)			123 (8.37)			39 (8.30)		
College degree or above	380 (90.26)			1,320 (89.80)			407 (86.60)		
Employment status									
Employed	270 (64.13)	1.933	0.164	508 (34.56)	3.524	0.060	236 (50.21)	2.489	0.115
Unemployed	151 (35.87)			962 (65.44)			234 (49.79)		
You or your relative or friend has experienced COVID-19									
Yes	38 (9.03)	0.156	0.693	108 (7.35)	0.008	0.930	60 (12.77)	0.499	0.480
No	383 (90.97)			1,362 (92.65)			410 (87.23)		
You or your relative or friend has participated in relevant work of prevention epidemic									
Yes	64 (15.20)	3.223	0.073	211 (14.35)	6.129	0.013	63 (13.40)	0.359	0.549
No	357 (84.80)			1,259 (85.65)			407 (86.60)		
You or your family member engaged in medical-related work									
Yes	37 (8.79)	1.340	0.247	151 (10.27)	1.518	0.218	51 (10.85)	3.103	0.078
No	384 (91.21)			1,319 (89.73)			419 (89.15)		
You were aware of public health before the COVID-19 outbreak									
Yes	97 (23.04)	0.420	0.517	316 (21.50)	4.950	0.026	109 (23.19)	3.969	0.046
No	324 (76.96)			1,154 (78.50)			361 (76.81)		

In the mixed-effect logistic regression analysis, Chinese residents who lived in urban (OR = 1.293, 95% CI = 1.051–1.591), “themselves or relative or friend has participated in relevant work of prevention epidemic” (OR = 1.553, 95% CI = 1.160–2.079), “themselves or family member engaged in medical-related work” (OR = 1.468, 95% CI = 1.048–2.056), and “were aware of public health before the COVID-19 outbreak” (OR = 1.428, 95% CI = 1.125–1.812) were more likely to support the promotion of public health education and training (Table 3).

**Table 3:** Mixed-Effect logistic regression analysis on the influencing factors of residents' attitudes of developing public health professionals.

**Variables**	**Coefficient**	***S.E*.**	***P***	***OR***	**95% *CI***
Gender (Ref: Female)					
Male	0.134	0.099	0.174	1.144	0.942–1.388
Age group, y (Ref: 18–44)					
45–59	−0.03	0.133	0.820	0.97	0.748–1.259
>60	0.216	0.215	0.315	1.242	0.814–1.894
Place of residence (Ref: Rural)					
Urban	0.257	0.106	0.015	1.293	1.051–1.591
Highest educational level (Ref: Primary school or below)					
Middle school	0.449	0.319	0.160	1.567	0.838–2.930
College degree or above	0.398	0.275	0.148	1.488	0.868–2.550
Region (Ref: Eastern China)					
Central China	−0.331	0.138	0.117	0.718	0.548–0.942
Western China	−0.177	0.164	0.279	0.837	0.607–1.155
Employment status (Ref: Unemployed)					
Employed	−0.076	0.11	0.492	0.927	0.748–1.150
You or your relative or friend has experienced COVID-19 (Ref: No)					
Yes	0.084	0.176	0.634	1.087	0.770–1.535
You or your relative or friend has participated in relevant work of prevention epidemic (Ref: No)					
Yes	0.44	0.149	0.003	1.553	1.160–2.079
You or your family member engaged in medical-related work (Ref: No)					
Yes	0.384	0.172	0.026	1.468	1.048–2.056
You were aware of public health before the COVID-19 outbreak (Ref: No)					
Yes	0.356	0.122	0.003	1.428	1.125–1.812

In addition, we stratified the study sample by regions and conducted multivariate logistic regression analyses. The results showed that for residents from Central China, “lived in urban” (Eastern China: OR = 1.951, 95% CI = 1.118–3.405), “has participated in relevant work of prevention epidemic” (Central China: OR = 1.560, 95% CI = 1.090–2.233), and “were aware of public health before the COVID-19 outbreak” (Central China: OR = 1.404, 95% CI = 1.045–1.887; Western China: OR = 1.831, 95% CI = 1.037–3.233) were the main factors associated with an increased willingness to support developing public health professionals (Table 4).

**Table 4:** Stepwise Multivariate Logistic Regression Analysis on the Influencing Factors of Residents' attitudes of developing public health professionals.

**Variables**	**Coefficient**	***S.E*.**	***P***	***OR***	**95% *CI***
**Eastern China**					
Place of residence (Ref: Rural)					
Urban	0.668	0.284	0.019	1.951	1.118–3.405
**Central China**					
You or your relative or friend has participated in relevant work of prevention epidemic (Ref: No)					
Yes	0.445	0.183	0.015	1.560	1.090–2.233
You were aware of public health before the COVID-19 outbreak (Ref: No)					
Yes	0.339	0.151	0.024	1.404	1.045–1.887
**Western China**					
You were aware of public health before the COVID-19 outbreak (Ref: No)					
Yes	0.605	0.290	0.037	1.831	1.037–3.233

## Discussion

In recent years, China has improved the quality of medical services and promoted the health of residents through vigorous reforms (22). However, more significant challenges remain, especially in the shortage of public health human resources during the COVID-19 outbreak in China, and a deeper reason, namely the weakness of public health education, also a common issue worldwide. The growing public awareness of the importance of public health following the COVID-19 outbreak will be an essential driver of policy for democratic governments. Public opinion on the development of public health education contributes to the formulation of health policy. The results of this study can be used as a reference for evidence-based health policy decision making, and play an innovative role in the future policy making of public health education.

Based on a crosssectional survey, this study determined the attitudes of Chinese residents toward developing public health professionals and influencing factors. We found that 74.50% of citizens supported the promotion of public health education and training in China, with only 22.11% of residents aware of public health before the COVID-19 outbreak. Moreover, this study had found some factors associated with the attitudes of Chinese residents of developing public health professionals, including those who lived in urban. These factors include “themselves or relative or friend has participated in relevant work of prevention epidemic,” “themselves or family member engaged in medical-related work,” and “were aware of public health before the COVID-19 outbreak.” They were more likely to support the promotion of public health education and training.

There is an obvious difference in the economic level between urban and rural areas in China. Urban residents are more willing to support the development of public health education, which may be due to their better living conditions. Previous studies have shown that the economic progress of a country can boost the health of its citizens (23, 24). For example, as the real GDP per capita of the world increased by 180% between 1970 and 2007 and infant mortality fell by 50% (25). The study of Jumbri et al. (26) also showed a link between economic status and health development. They found that residents of areas with better economic conditions are more likely to pursue high-quality health, which is yet another piece of evidence of the relationship between economics and health.

People who themselves, or whose relative or friend has participated in relevant work of epidemic prevention, as well as those who themselves or who have a friend or family member engaged in medical-related work also expressed sufficient support for public health education. This support may come from their personal feelings based on their education and experiences. Personal perceptions were significantly associated with policy support. As health literacy increases, support generally increases, similar to findings in Julia et al. (27) and Bhawra et al. (28). Therefore, health policymakers should choose to enact policies when public perception is most potent, such as implementing public health policies in the aftermath of the COVID-19 outbreak.

Participants who were aware of public health before the COVID-19 outbreak will expect the development of public health education, which means that the more people know about public health, the better will be the development of it. Previous analysis has shown that effective policy actions promote policy understanding from the masses and are consistent with the behavioral, socio-economic, and demographic characteristics of the people they seek support (29–31). Public understanding plays a fundamental role in implementing policies (32, 33). After the COVID-19 pandemic, increased awareness of the importance of public health among the general public will facilitate the implementation of relevant initiatives.

This study found that economic status, personal perception, and understanding are the crucial factors that influence the support of the public for the development of public health education. These factors will drive public opinion and ultimately influence China's public health development and medical reform in the future. The COVID-19 pandemic has far-reaching implications for human society, and in fact, this impact will feed into future policies based on public opinion. Public opinion will play an important role in the formulation and implementation of public health education policies in the future.

### Strengths and Limitations

The present study is the first to discuss the impact of COVID-19 on public opinion and public health education. We used a nationwide sample of the Chinese population. The perspective of this study can provide some reference for future research on public policy theory, and help researchers better understand the process of health policy formation.

However, this study has some limitations. First, this study used social media as the main method to disseminate the survey. Participants without access to the internet were probably not included. Second, the distribution of the study participants was imbalanced across regions (421:1,470:470); therefore, the subgroups of variables might not be representative of the population. Third, this study could not determine how many participants reviewed the online poster or survey but decided not to complete the survey; thus, the presence of non-response bias could not be assessed. Fourth, there is no occupational breakdown of the participants, which could cause bias by occupation factors. Finally, as the behaviors were self-reported, reporting bias was possible. Overall, the generalization of the results should be regarded with caution.

Future research could explore more factors that may influence the options of the residents based on the present study, such as major, occupation, or social culture. In addition, longitudinal studies should be conducted in the future to evaluate the relationship between various influencing factors and attitudes of developing public health professionals among residents.

## Conclusions

This study found that 74.50% of citizens supported the promotion of public health education and training in China, with economic status, personal perception, and understanding being the important factors that influence public opinion. COVID-19 has aroused the attention of Chinese residents to public health education, as only 22.11% of residents were aware of public health before the COVID-19 outbreak. The COVID-19 pandemic has far-reaching implications for human society, and in fact, this impact will feed back into future public health policies based on public opinion.

## Data Availability Statement

The raw data supporting the conclusions of this article will be made available by the authors, without undue reservation.

## Ethics Statement

The studies involving human participants were reviewed and approved by this study protocol was approved by the institutional review board of Tongji Medical College of Huazhong University of Science and Technology, Wuhan, China. All methods were performed in accordance with the relevant guidelines and regulations. Respondents were informed that their participation was voluntary, and consent was implied on the completion of the questionnaire. The patients/participants provided their written informed consent to participate in this study.

## Author Contributions

XS, CJL, TD, and YG conceived and designed the study. JF, HC, ZL, and ZW participated in the acquisition of data. XS and CJL analyzed the data. CL and XH gave advice on methodology. XS and TD drafted the manuscript. YG and XS revised the manuscript. YG is the guarantor of this work and had full access to all the data in the study and takes responsibility for its integrity and the accuracy of the data analysis. All authors read and approved the final manuscript.

## Conflict of Interest

The authors declare that the research was conducted in the absence of any commercial or financial relationships that could be construed as a potential conflict of interest.

## Publisher's Note

All claims expressed in this article are solely those of the authors and do not necessarily represent those of their affiliated organizations, or those of the publisher, the editors and the reviewers. Any product that may be evaluated in this article, or claim that may be made by its manufacturer, is not guaranteed or endorsed by the publisher.
